# Glass Formation Area and Structural Analysis of the Rich TeO_2_ Zone in the CdO–TeO_2_–GeO_2_ System

**DOI:** 10.1021/acsomega.5c12889

**Published:** 2026-06-09

**Authors:** David Alejandro Rodríguez Carvajal, María Elena Zayas Saucedo, Iveth Viridiana García Amaya, José Jesús Cayetano Borquez Gamboa, Lamberto Castro Arce, José Manuel Cortez Valadez

**Affiliations:** † Universidad de Sonora, Departamento de Investigación en Física, Blvd. Luis Encinas y Rosales S/N, Col. Centro, Hermosillo, Sonora CP 83000, México; ‡ Universidad Estatal de Sonora, Ingeniería en Biomédica, Av. Ley Federal del Trabajo S/N, Hermosillo, Sonora 83100, México; § Universidad Estatal de Sonora, Ingeniería en Geociencias, Av. Ley Federal del Trabajo S/N, Hermosillo, Sonora 83100, México; ∥ Universidad de Sonora, Departamento de Física, Matemáticas e Ingeniería, Lázaro Cárdenas del Río 100, Francisco Villa, Navojoa, Sonora 85880, México; ⊥ 27813 SECIHTI-Universidad de Sonora, Departamento de Investigación en Física, Blvd. Luis Encinas y Rosales S/N, Col. Centro, Hermosillo, Sonora CP 83000, México

## Abstract

This study systematically explored the glass formation region of the ternary CdO–TeO_2_–GeO_2_ glass system using the conventional melt-quenching method. Thirty-six compositions with varying TeO_2_ contents and relative CdO proportions were synthesized at melting temperatures of 900 °C–1200 °C, revealing a broad glass formation region that included fully transparent and partially crystallized glasses. The structural and thermal properties of representative TeO_2_-rich compositions were investigated. X-ray diffraction analysis confirmed predominantly amorphous structures across most of the investigated compositional range, while weak diffraction peaks were observed in compositions with the highest CdO contents, indicating the onset of crystallization; besides, the S26 sample showed three characteristic peaks corresponding to Cd_3_TeO_6_. Energy-dispersive X-ray spectroscopy confirmed Cd, Te, and Ge as the main glass constituents. Observed deviations from nominal compositions were attributed to Te volatilization, elemental redistribution during melting, and partial Al incorporation from the alumina crucible. Raman and Fourier-transform infrared spectroscopic results revealed the progressive depolymerization of the tellurite network with increasing CdO content, which was associated with the transformation of TeO_4_ units into TeO_3+1_ polyhedra and TeO_3_, together with changes in the coordination environment of Ge ions, suggesting the coexistence of GeO_4_ and GeO_6_ units. Differential scanning calorimetry revealed glass transition temperatures of 416 °C–495 °C. CdO-rich compositions exhibited multiple crystallization events, whereas TeO_2_-rich compositions remained amorphous up to 800 °C, indicating that CdO promotes devitrification, whereas GeO_2_ enhances thermal stability.

## Graphical abstract

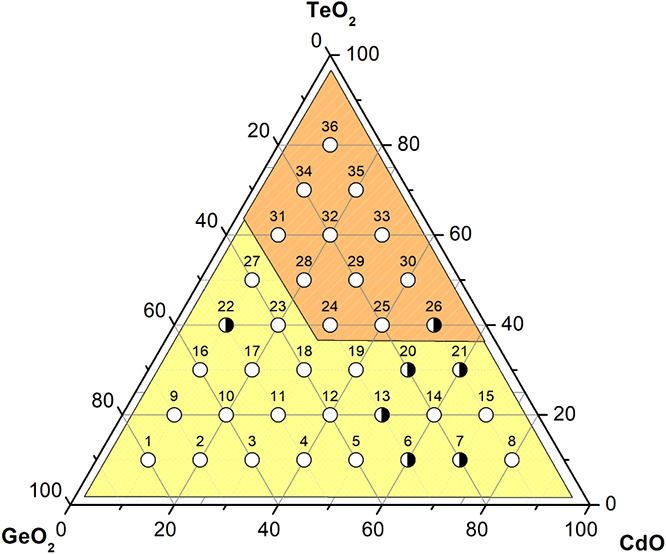

## 1 Introduction

Oxide glasses continue to attract substantial research interest owing to their versatility in optical and photonic applications, particularly in the ultraviolet (UV), visible, and near-infrared (NIR) spectral regions. Among these materials, glasses based on tellurite (TeO_2_) and germanate (GeO_2_) stand out for their favorable optical characteristics, including a high refractive index, wide transparency window, and good thermal and chemical stability, making them suitable for applications such as optical fibers, laser devices, nonlinear optics components, and spectroscopy.^1–4^ Consequently, TeO_2_–GeO_2_ glass systems have been successfully explored as host matrices for rare-earth ions and in applications such as broadband optical amplifiers and optical filters.^5–7^ TeO_2_ is well-known for its low phonon energy and high refractive index, which are advantageous for minimizing nonradiative losses and improving optical efficiency.^8^ However, glasses based solely on TeO_2_ often exhibit limited thermal stability. In this context, the incorporation of GeO_2_ has been reported to strengthen the rigidity and stability of the glass network, leading to higher glass transition temperatures (*T*_g_) and enhanced durability. Beyond the selection of glass formers, the addition of network modifiers plays a crucial role in tailoring the physical and optical properties of oxide glasses. For example, heavy metal oxides are known to increase density, the refractive index, and infrared transmission.^9^ Cadmium oxide (CdO) is of particular interest among these modifiers owing to its high electronic polarizability and partially covalent bonding character.^10,11^ Unlike conventional alkali or alkaline-earth oxides, CdO can influence the structural arrangement of oxide glasses by potentially modifying the coordination environment, contributing to changes in their glass formation, thermal behavior, and stability.^12^

Several studies have examined the CdO–TeO_2_–GeO_2_ ternary system and have shown that high CdO contents may promote the formation of inverted glass structures, accompanied by changes in the coordination of Cd^2+^ ions between tetrahedral and octahedral environments.^13^ These observations suggest that CdO may behave not only as a network modifier but also, depending on composition, as an intermediate oxide. In addition, this glass system has been investigated as a host matrix for rare-earth ions, exhibiting promising optical activation for applications such as phosphors and near-ultraviolet (NUV)-based white light-emitting devices.^14^ Other studies have reported the occurrence of CdTe and aluminum germanate nanocrystalline phases in glasses belonging to the CdO–TeO_2_–GeO_2_ system. In particular, the formation of aluminum-containing phases has been attributed to aluminum incorporation resulting from crucible corrosion during melting, likely favored by the chemical aggressiveness of the glass batch and the high reactivity of the melt under the synthesis conditions.^15^ Despite these contributions, a comprehensive understanding of the glass-forming region is still lacking, especially regarding the interplay among glass-forming ability, structural features, and thermal stability.

In this work, a systematic investigation of the glass-forming region in the CdO–TeO_2_–GeO_2_ ternary system is presented, with special emphasis on the TeO_2_-rich compositional domain. The study aims not only to delimit the range of compositions capable of forming fully amorphous glasses, but also to establish correlations among composition, structural organization, and thermal behavior. Particular attention is devoted to the comparison between TeO_2_-rich glasses and compositions with higher CdO contents. On the basis of this compositional survey, a representative region suitable for detailed structural characterization was identified and examined by X-ray diffraction, energy-dispersive X-ray spectroscopy, FT-IR, and Raman spectroscopies, while differential scanning calorimetry was employed to evaluate the thermal stability of the selected glasses.

## 2 Experimental Section

Thirty-six CdO–TeO_2_–GeO_2_ compositions were fabricated for glass formation region analysis, among which 12 representative TeO_2_-rich compositions were selected for characterization. For each composition, 10 g batches were prepared using high-purity CdO, TeO_2_, and GeO_2_ powders (Sigma-Aldrich, 99.99% purity), which were homogenized prior to melting in high-alumina crucibles within a Thermolyne 46100 furnace. Figure 1 presents a Gibbs triangle of the 36 compositions. The 12 selected TeO_2_-rich compositions are listed in Table 1. The samples were labeled according to wt % their nominal composition in of CdO, TeO_2_, and GeO_2_ powders. Owing to the markedly different melting points and volatilization behaviors of TeO_2_, CdO, and GeO_2_, the synthesis temperature was adjusted depending on the composition to obtain a homogeneous melt (Table 1). While TeO_2_-rich compositions could be vitrified at 900 °C, glasses with a higher GeO_2_ content required substantially higher melting temperatures, in some cases 1200 °C. Vitrification was achieved via rapid quenching onto a stainless-steel mold, followed by annealing at 300 °C for 2 h, a practice commonly adopted in TeO_2_ glass systems to reduce residual thermal stresses,^16–19^ although complete stress relaxation may not have been achieved due to the annealing temperature being significantly below *T*_g_.

**Figure 1:**
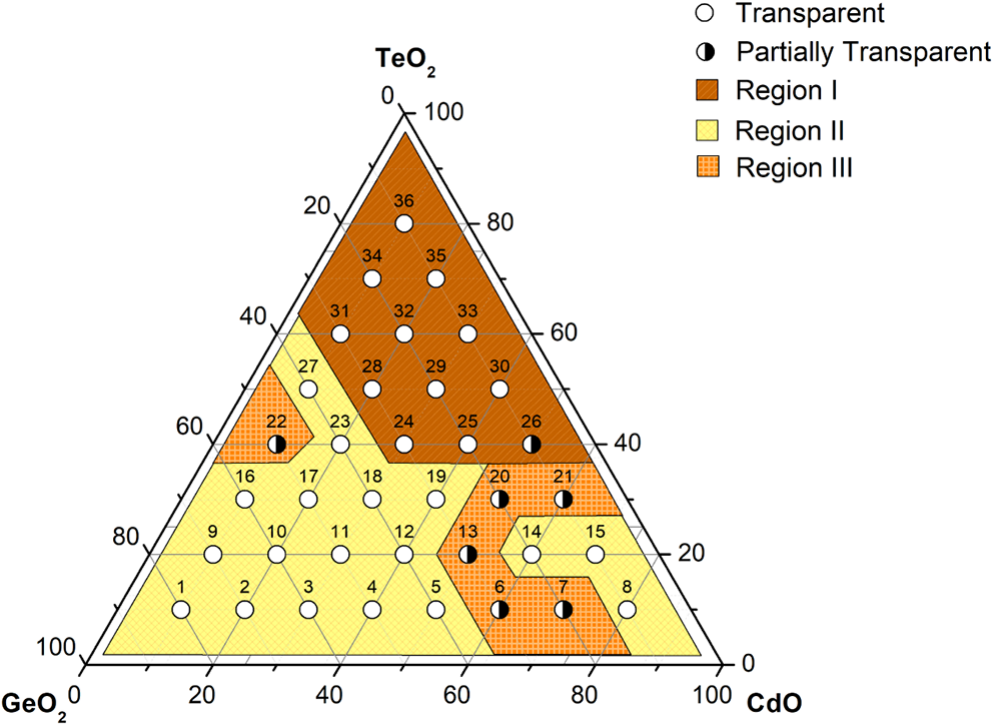
Gibbs triangle showing the formation region of transparent and partially crystallized glasses (wt %). Region I corresponds to TeO_2_-rich glasses (object of study), Region II to transparent glasses outside the scope of this work, and Region III to partially crystallized glasses.

**Table 1:** Compositions in wt % and mol % Object of Study

	CdO		TeO_2_		GeO_2_		Melting
Sample	wt %	mol %	wt %	mol %	wt %	mol %	°C
**S36**	10	11.54	80	74.29	10	14.17	900
**S35**	20	22.57	70	63.58	10	13.85	950
**S34**	10	11.0	70	61.99	20	27.01	1000
**S33**	30	33.13	60	53.32	10	13.55	1000
**S32**	20	21.55	60	52.01	20	26.44	1100
**S31**	10	10.52	60	50.77	30	38.71	1200
**S30**	40	43.24	50	43.49	10	13.27	1000
**S29**	30	31.65	50	42.45	20	25.9	1100
**S28**	20	20.61	50	41.45	30	37.94	1200
**S26**	50	52.93	40	34.08	10	12.99	1100
**S25**	40	41.35	40	33.27	20	25.38	1150
**S24**	30	30.3	40	32.51	30	37.19	1200

Subsequently, the glasses were crushed and sieved to obtain particles of approximately 30 μm. X-ray diffraction (XRD) patterns were recorded using a Bruker D8 Advance diffractometer with Cu Kα radiation. Energy-dispersive X-ray spectroscopy (EDS) was performed using a JEOL JSM-series scanning electron microscope. Raman spectra were acquired using a LabRam HR Jobin-Yvon-Horiba spectrometer equipped with a He–Ne laser (632.8 nm). Fourier-transform infrared (FT-IR) spectra were collected using a PerkinElmer 1600 series spectrometer within the 4000–400 cm^–1^ range. Differential scanning calorimetry (DSC) measurements were performed on powdered glass samples using a TA Instruments SDT 2960 analyzer. A 20 mg sample of glass powder was heated from 20 to 1100 °C at 10 °C/min on a 20 mg bed of aluminum oxide (Al_2_O_3_) under a nitrogen atmosphere.

## 3 Results and Discussion

### 3.1 Glassy Phase Formation

The investigated members of the CdO–TeO_2_–GeO_2_ glass system family included transparent and partially crystallized glasses. The melting behavior of the samples reflected the characteristic high volatility and low viscosity of this system, wherein the specific behaviors of CdO and TeO_2_ substantially influenced the final glass composition. Within TeO_2_–GeO_2_ glass systems, CdO is known to act as an intermediate structural unit, with the composition strongly affecting the coordination environment and effect on melt properties.

Figure 1 presents the glass formation region of the CdO–TeO_2_–GeO_2_ system. Two types of glasses with different TeO_2_ and GeO_2_ contents were identified. Region I corresponded to visually homogeneous and predominantly transparent glasses, except for sample 26, which exhibited a translucent visual appearance. Region III corresponded to glasses exhibiting partial opacity or visible surface heterogeneities. These regions represented 19 samples, 6 of which were partially crystallized: samples S6 (60–10–30), S7 (70–10–20), S13 (50–20–30), S20 (50–30–20), S21 (60–30–10), and S22 (10–40–50). Within the compositional series, samples with nominal compositions of 60–10–30 (57.2–7.7–35.1 mol %), 70–10–20 (68.2–7.9–23.9 mol %), and 60–30–10 (62.2–25.0–12.8 mol %) were categorized as inverted glasses. According to the literature, this designation is generally applied to compositions in which the content of CdO surpasses those of TeO_2_ and GeO_2_.^20^

TeO_2_-rich glasses exhibited a visually homogeneous appearance and coloration that ranged from colorless to pale yellow, yellow, brown–orange, and green–yellow Figure 2. This feature did not display an evident compositional dependence, as coloration varied across the investigated compositional range. In the literature, coloration is associated with the partial reduction of Te^4+^ species to metallic Te^0^ during the melting process.^21,22^ This reduction can be promoted by local processing conditions, including high melting temperatures, TeO_2_ volatilization, and transient redox environments within the melt. Although metallic Te^0^ was not directly identified in this study, the recurrent appearance of coloration suggested that redox effects contributed to the observed glass formation behavior.^23^ This effect is likely related to complex redox equilibria established within the melt, which may be frozen in different states during cooling, thereby favoring the development of dark colors.^22^

**Figure 2:**
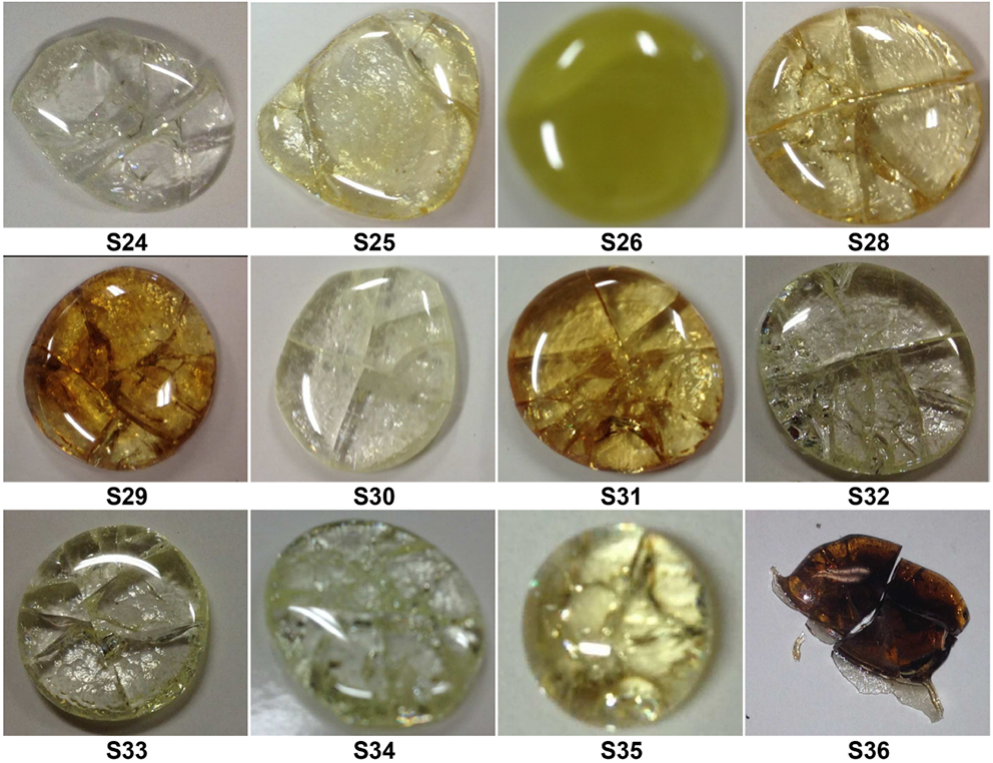
Photograph of the studied glass samples illustrating their appearance.

### 3.2 XRD and EDS

The structural characteristics of the glasses located in Region I were investigated via XRD, as shown in Figure 3. All the XRD patterns, revealed a broad diffraction band centered at 2θ = 27°–30° that was indicative of a lack of long-range order.^24^ It should be noted that the center of the broad band shifts toward lower angles with increasing TeO_2_ content. The XRD pattern of sample S26 exhibited a relatively more intense diffraction peak at 2θ = 31.7°, 31.8° and 32.5°, which are consistent with the three principal peaks of PDF#76–1007, assigned to the Cd_3_TeO_6_ phase. Conversely, the XRD pattern of sample S30 exhibited a single weak diffraction peak located at 2θ = 33.1°, suggesting the onset of incipient crystallization.^25^ Both samples possessed the highest CdO contents within the compositional series. Notably, in the TeO_2_-rich region of the Gibbs triangle, samples S26 and S30 were the only compositions that exhibited slightly crystalline characteristics.

**Figure 3:**
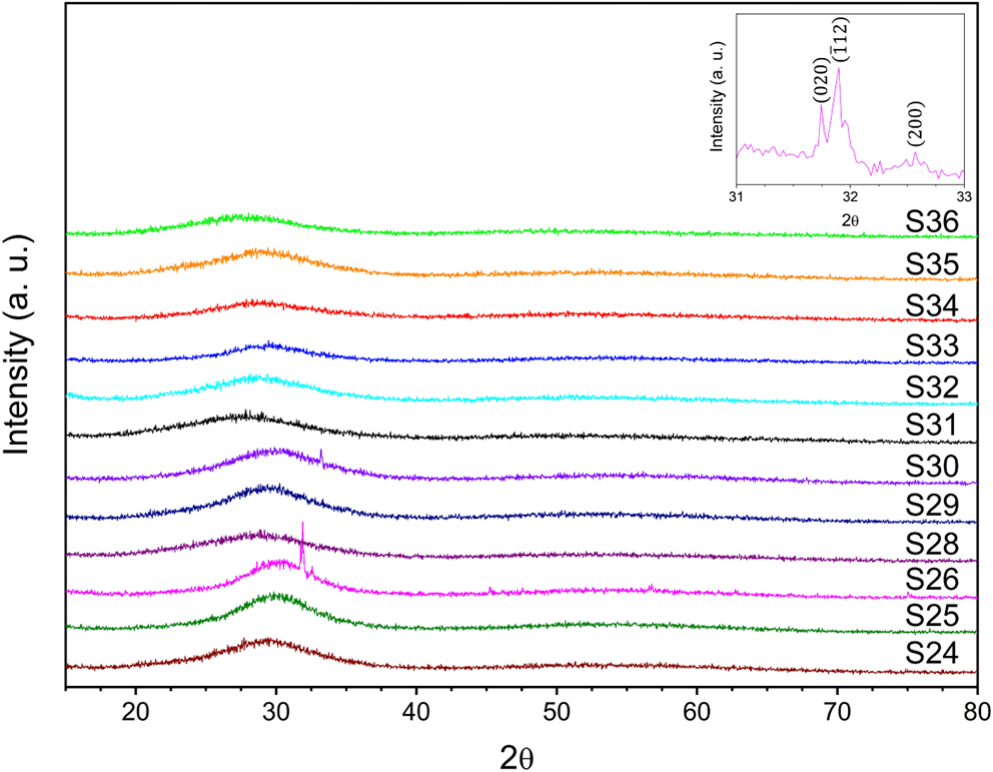
X-ray diffraction (XRD) patterns of glasses from Region I.

Elemental analysis of the CdO–TeO_2_–GeO_2_ glass system was performed by energy-dispersive X-ray spectroscopy (EDS), and the results are summarized in Table 2. Seven representative compositions (S24, S25, S26, S28, S30, S32 and S36) were examined in order to verify elemental incorporation and compositional trends across the series.

**Table 2:** EDS of Chemical Compositions in wt % for Selected Glasses

Sample	CdO	TeO_2_	GeO_2_	Al_2_O_3_
**S24**	28.19	37.88	33.93	0.00
**S25**	38.90	39.80	21.30	0.00
**S26**	49.00	42.36	6.50	2.14
**S28**	23.30	44.00	32.70	0.00
**S30**	35.90	48.05	12.07	3.98
**S32**	21.50	51.40	27.10	0.00
**S36**	11.74	71.41	8.22	8.63

EDS analysis confirmed that Cd, Te, and Ge were the main constituent elements in all samples, although notable deviations from nominal compositions were observed. These differences suggested that the final chemical composition of the glasses was affected by compositional redistribution during melting, possible volatilization phenomena, and, in some cases, contamination derived from the alumina crucible.^23,24,26^ Samples S24 and S25 exhibited relative Ge enrichment, suggesting partial Te loss during melting or a local compositional heterogeneity that favored Ge-rich domains. A similar trend was observed in samples S28 and S32, where the Cd and Ge contents were slightly higher than expected, while the Te content was reduced. Given that TeO_2_ is known to be more volatile than GeO_2_ under high-temperature processing conditions, the decreased Te content in these samples may have been associated with partial Te loss during melting. Based on the comparison between the initial compositions and the EDS results, it was estimated that, on average, approximately 3.5 wt % (3.97 mol %) of TeO_2_ is lost due to volatilization.

By contrast, sample S26 exhibited a higher Te content and lower Ge content than expected, together with 2.14 wt % Al_2_O_3_, suggesting that, in addition to local compositional redistribution, some degree of alumina incorporation occurred during melting, likely due to interaction with the crucible. The same phenomenon was evident in samples S30 and S36, where the Al_2_O_3_ contents reached 3.98 and 8.63 wt %, respectively. The detection of aluminum (Al) in these samples strongly supported crucible-derived contamination, which may have modified the melt chemistry and influenced the final structure of the glasses.^26^ Importantly, no crystalline phases containing Al were detected via XRD, and no vibrational features attributable to Al–O structural units were identified via Raman spectroscopy.^22,27^ Sample S30 exhibited Cd and Te losses, accompanied by Ge enrichment and Al incorporation. This behavior may have been related to the combined effect of volatilization of more unstable components and local retention of Ge-rich domains. Sample S36, which possessed the highest nominal composition of TeO_2_, exhibited marked Te and Ge losses and a substantial Al contribution, suggesting that this composition was particularly susceptible to melt–crucible interaction during processing.

Overall, the EDS results indicated that the final compositions of the glasses did not exactly reproduce the initial batch stoichiometry. The main factors responsible for these deviations were likely: (i) preferential volatilization of Te-containing species, (ii) local elemental redistribution within the melt, and (iii) contamination derived from the alumina crucible. These compositional variations may have contributed to the structural variations and crystallization behaviors observed via XRD, especially in samples with a higher CdO content or detectable Al incorporation.

### 3.3 Raman Spectroscopy

Raman spectroscopy was performed to gain deeper insight into the structural characteristics of the CdO–TeO_2_–GeO_2_ glass system, and the spectra of all investigated samples are presented in Figure 4. The Raman spectra of all glasses exhibited two broad and well-defined bands centered at approximately 300–600 and 600–900 cm^–1^. The band within the 400–600 cm^–1^ range was attributed to Te–O–Te linkages connecting TeO_4_ and TeO_3+1_ units, while the band at 600–790 cm^–1^ was associated with Te–O^–^ stretching vibrations in TeO_4_, TeO_3+1_, and TeO_3_ structural units. The latter band shifted toward higher wavenumbers with increasing CdO content (from 741 to 760 cm^–1^), indicating that the incorporation of CdO induced a transformation in the Te^4+^ coordination environment from TeO_4_ to TeO_3_ units.^28^ TeO_4_ units underwent depolymerization with the progressive addition of CdO, forming TeO_3_ trigonal pyramidal and TeO_3+1_ species containing elongated Te–O_ax_ bonds. This structural rearrangement was supported by the increased intensity of the 600–790 cm^–1^ band in glasses with the highest CdO contents.

**Figure 4:**
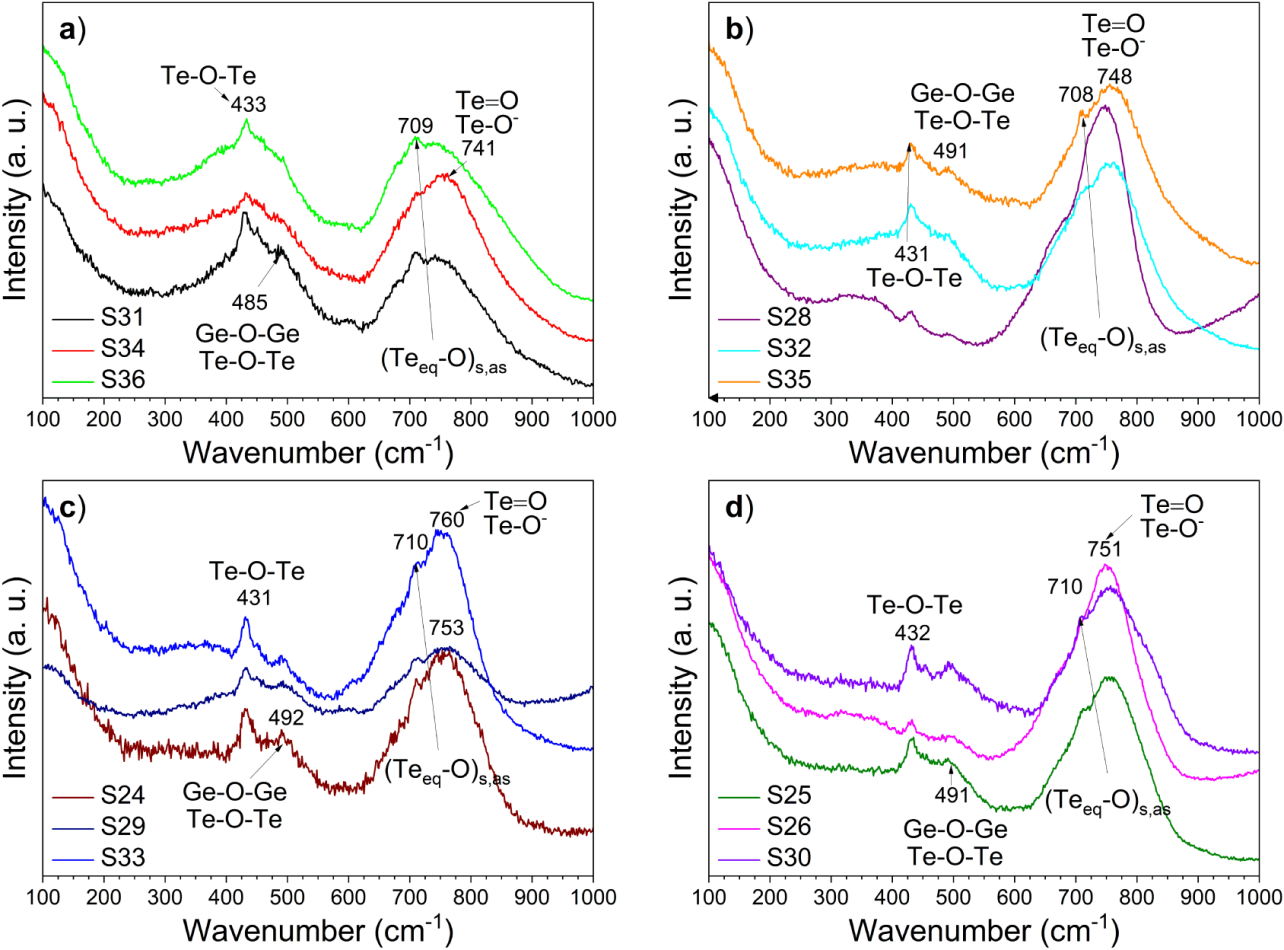
Raman spectra for the glasses considered in this study. a) S31, S34, S36; b) S28, S32, S35; c) S24, S29, S33 and d) S25, S26, S30.

The band at 431–433 cm^–1^ was mainly attributed to the symmetric stretching and/or bending vibrations of Te–O–Te linkages involving corner-sharing structural units,^29^ although a contribution from symmetric Ge–O–Ge vibrations in four-membered GeO_4_ rings could not be ruled out.^29^ This feature is associated with bridging-oxygen linkages, thereby providing information on the connectivity of the glass network.^30^ As shown in Figures 4(a) and 4(b), this band was more intense for glasses containing 10 wt % CdO compared to those containing 20 wt % CdO, although a consistent trend was not observed for glasses with even higher CdO contents [Figures 4(c) and 4(d)].

The band observed at approximately 485–492 cm^–1^ suggested the participation of TeO_2_ and GeO_2_ structural vibrations. This band can be associated with Ge–O–Ge linkages in three-membered rings built from GeO_4_ tetrahedra, while Te–O–Te bending vibrations may also be present in this spectral region.^6^ An increase in the intensity of this band indicated that these structural arrangements became more relevant in several compositions; however, this behavior did not follow a strictly proportional trend with TeO_2_ content. At the highest TeO_2_ content, the disappearance or weakening of this feature suggested that the network became dominated by TeO_2_-related linkages, which masked or reduced the contribution of GeO_2_ ring vibrations. Meanwhile, a small peak appeared at 708–710 cm^–1^ on the band centered at 760 cm^–1^, which has been reported as the stretching vibrations of (Te_eq_–O)_s_ and (Te_eq_–O)_as_ in TeO_3+1_ polyhedra or TeO_3_ trigonal pyramidal units with a possible contribution from deformation modes of Ge atoms in the glassy network.^31–35^

The 741–760 cm^–1^ band, which was the most intense feature across all samples, was attributed to the stretching modes of Te–O^–^ and Te=O bonds associated with nonbridging oxygens (NBOs) in TeO_3_ trigonal pyramidal and TeO_3+1_ units.^32,33,35^ This band is typically absent in pure TeO_2_, and its presence indicated the cleavage of Te–O–Te linkages within the initially polymerized network due to the incorporation of CdO. Consequently, TeO_4_ units were progressively transformed into TeO_3+1_ polyhedra and TeO_3_ units containing NBOs.

Taken together, the observed shifts in Raman features reflected a structural evolution from a TeO_2_-rich network to the formation of Cd_*x*_Te_*y*_O_*z*_ compounds comprising TeO_3_ units.^36^ Although minor crystalline features were observed via XRD analysis in samples S26 and S30, their Raman spectra remained dominated by broad bands typical of an amorphous network, indicating that the crystalline fraction was limited. Notably, no Raman bands corresponding to Te–Te vibrational modes were observed in the low-frequency region, suggesting the absence of detectable metallic Te^0^ species.^22,27^ Therefore, the observed coloration was attributed to structural and compositional variations within the TeO_2_ network rather than to metallic Te^0^ formation, since this species is typically confirmed in Raman spectroscopy by well-defined peaks or bands in the 85–135 cm^–1^ range.^22^

From a structural classification perspective, the Raman spectroscopic results indicated that CdO predominantly acted as a network modifier within the investigated compositional range, promoting NBO formation and inducing depolymerization of the TeO_2_ network. Nevertheless, in highly modified compositions, Cd^2+^ exhibited intermediate characteristics, strongly interacting with the surrounding oxygen polyhedra rather than behaving as a simple ionic modifier. Similar compositional dependence has been reported in related TeO_2_ and TeO_2_–GeO_2_ glass systems.^11,13,20^ Collectively, results indicated that while CdO mainly behaved as a network modifier in the investigated glasses, its structural role remained composition dependent.

### 3.4 Infrared Spectroscopy

The infrared spectra of the investigated samples are presented in Figure 5. As shown in Figures 5(a) and 5(b), a broad absorption envelope was observed over the 1000–450 cm^–1^ range, whereas the main absorption region extended from 900 to 500 cm^–1^ [Figures 5(c) and 5(d)]. The broad bands centered between 715 and 641 cm^–1^ were attributed to the vibrations of TeO_3_/TeO_4_ structural units.^37–39^ By contrast, the shoulder located at higher wavenumbers (820–795 cm^–1^) was assigned to GeO_4_ units.^38^ Notably, the intensity of this shoulder progressively decreased and eventually disappeared with increasing TeO_2_ content. As shown in Figure 5(a), the band at 715 cm^–1^ shifted to 700 cm^–1^ with increasing TeO_2_ content, suggesting a change in the local environment of Te atoms, likely due to the increased formation of Te–O–Te bridges and conversion from TeO_4_ (distorted trigonal bipyramids) to TeO_3_ (trigonal pyramids) units as the polyhedra became longer and more polarized.^34,39^ The band near 715 cm^–1^ is commonly associated with the NBO stretching modes of TeO_3_ structures.^31^ Given that the CdO content remained constant in this subset, the observed shift was mainly attributed to TeO_2_-driven structural reorganization. Meanwhile, the second band shifted from 820 to 815 cm^–1^ with decreasing GeO_2_ content, which was consistent with its assignment as the asymmetrical stretching vibrations of Ge–O–Ge bonds forming connections with GeO_4_ units.^38^

**Figure 5:**
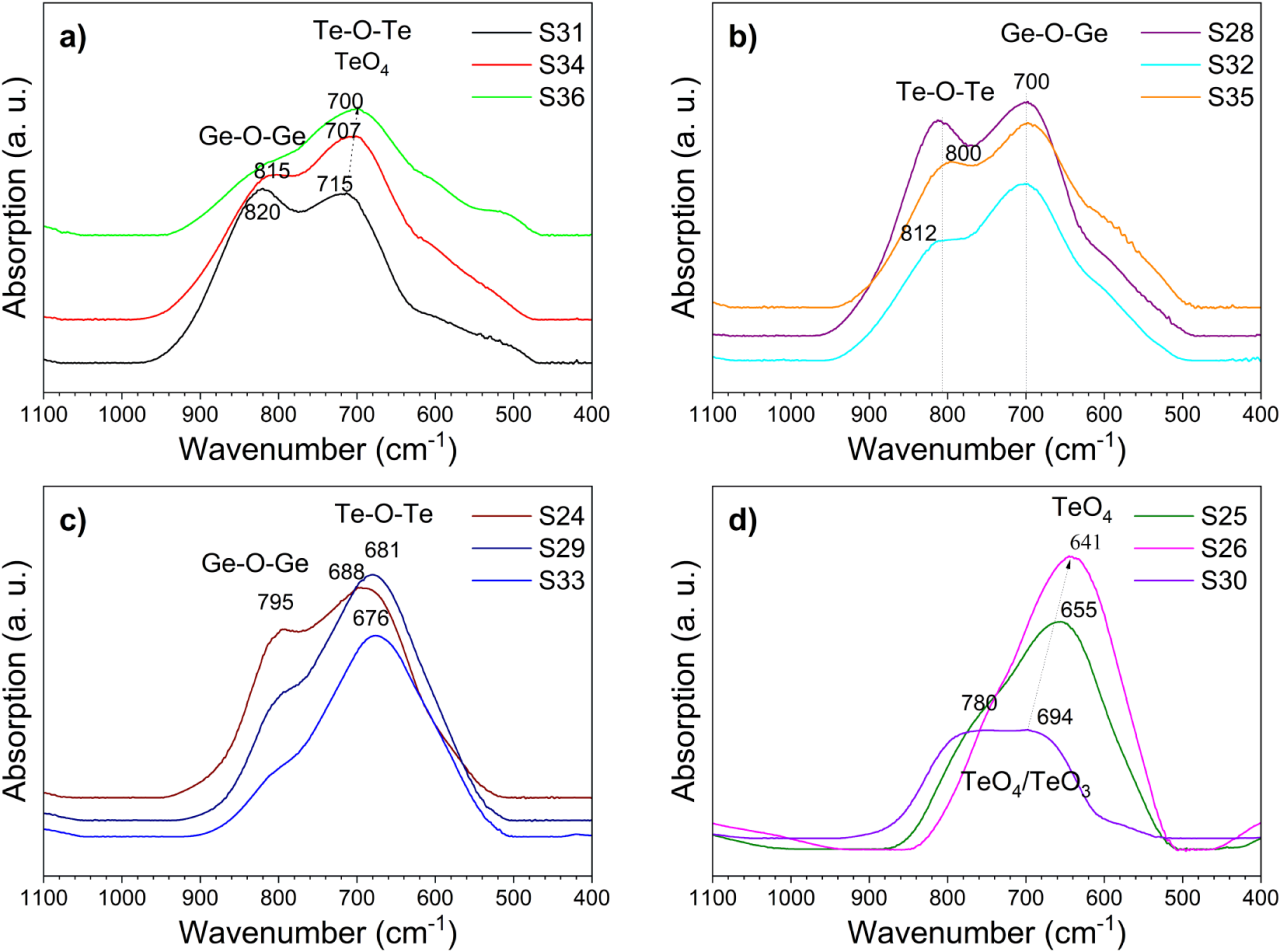
FT-IR spectra of the glasses under study. a) S31, S34, S36; b) S28, S32, S35; c) S24, S29, S33 and d) S25, S26, S30.

As shown in Figure 5(b), the band at 700 cm^–1^ remained constant despite increasing TeO_2_ content. The CdO content was slightly higher (20 wt %) for these samples, which may have enhanced its influence on the local glass structure. Cd^2+^ likely promoted NBO formation and an early depolymerization of the tellurite network.^40^ As a result, the structural environments associated with this band may already be established, leading to the absence of a significant spectral shift upon further TeO_2_ addition. In contrast, the second band shifts toward lower wavenumbers as the GeO_2_ content decreases.

Figure 5(c) illustrates that the first band shifted from 688 to 676 cm^–1^ with increasing TeO_2_ content, demonstrating a behavior similar to that observed in the first set of samples. Meanwhile, the band at 795 cm^–1^ gradually faded with decreasing GeO_2_ content. The opposite behavior is illustrated in Figure 5(d): the band at 694 cm^–1^ shifted to lower wavenumbers with decreasing TeO_2_ content. This shift likely reflected a weakened Te–O bonding environment due to the higher CdO content and lower TeO_2_ content, which reduced the network-forming ability of the system. Meanwhile, Cd^2+^ broke down Te–O–Te bridges, increasing the abundance of NBOs and disrupting connectivity. The band shift to lower frequencies (∼641 cm^–1^) suggested the formation of a less constrained Te–O environment, possibly indicative of the presence of more TeO_3_-like units, which vibrated at lower frequencies than TeO_4_ units.^34^

### 3.5 DSC

The DSC thermograms revealed that the thermal stability and devitrification tendency of the CdO–TeO_2_–GeO_2_ glass system strongly depended on the relative CdO and GeO_2_ contents. In general, compositions with a higher CdO content exhibited more pronounced exothermic events, whereas those with a lower CdO content and/or a higher GeO_2_ content displayed a smoother thermal response or even no detectable crystallization within the analyzed temperature range, as shown in Figure 6. This behavior was consistent with the structural role of each oxide: CdO acts as a network modifier and promotes depolymerization, while GeO_2_ strengthens the connectivity of the glass network.^41–43^

**Figure 6:**
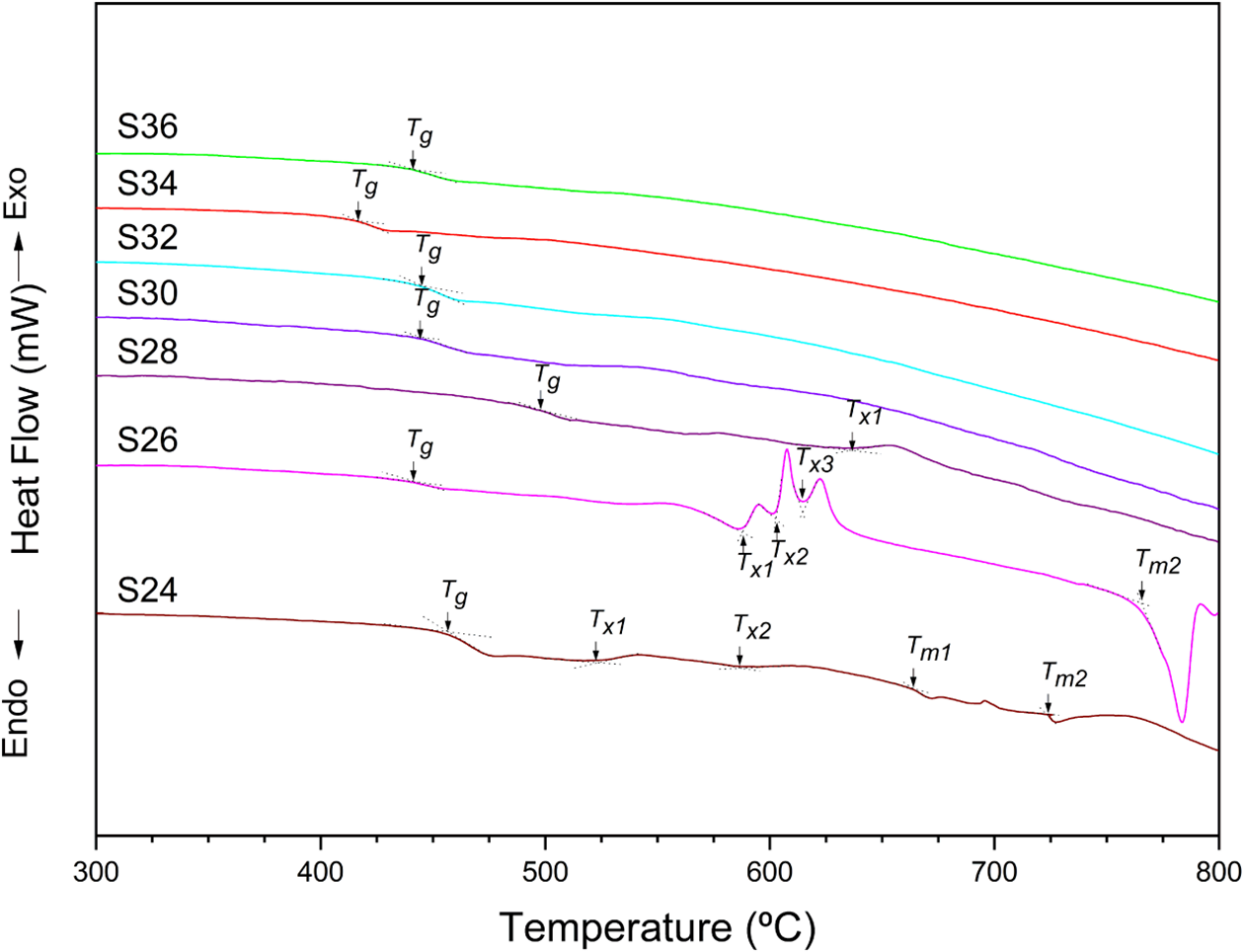
DSC thermograms of the glasses under study.

In Table 3, the thermal events detected in the samples analyzed by DSC are presented. Sample S24 exhibited a *T*_g_ value of 458 °C, two crystallization events at *T_x_*_1_ = 521 °C and *T*_*x*2_ = 587 °C, two endothermic events at *T*_*m*2_ = 663 °C and *T*_*m*2_ = 723 °C, and Δ*T* = 63 °C. The relatively low Δ*T* value indicated that devitrification began at a temperature relatively close to *T*_g_, suggesting the moderate thermal stability of the sample. The presence of two exothermic and two endothermic events pointed to a stepwise crystallization process followed by the melting of more than one crystalline fraction or phases with different thermal stabilities.^44–46^ Given that this composition contained equivalent proportions of CdO and GeO_2_ (30 wt % each), the observed thermal behavior suggested a sequential crystallization process in which the first exothermic event associated with initial nucleation and the second associated with crystal growth or transformation of a previously formed phase. The endothermic events at 663 and 723 °C likely corresponded to the melting of these crystallized phases.

**Table 3:** Transition Temperatures, Exothermic and Endothermic Peaks (DSC)

Sample	*T*_g_ (°C)	*Tx*_1_ (°C)	*Tx*_2_ (°C)	*Tx*_3_ (°C)	*Tm*_1_ (°C)	*Tm*_2_ (°C)	Δ*T* (°C)
**S24**	458	521	587		663	723	63
**S26**	442	587	603	614	772		146
**S28**	445	544					99
**S30**	495	563	634				69
**S32**	445						
**S34**	442						
**S36**	416						

Sample S26 exhibited a *T*_g_ value of 442 °C, three well-defined exothermic peaks at 587 °C, 603 °C, and 614 °C, one endothermic event at 772 °C, and the highest Δ*T* value of 146 °C among the crystallized samples. This behavior suggested that, although crystallization began well above *T*_g_, an intense sequence of devitrification processes occurred upon reaching the activation temperature. This composition had the highest CdO content and one of the lowest GeO_2_ contents within the compositional series, making it structurally the most prone to glass network depolymerization and the generation of a favorable environment for the precipitation of Cd-rich crystalline phases. Based on recent results reported for the similar system, where CdO and Cd_3_TeO_6_ were detected in CdO-rich compositions,^47^ the three exothermic peaks were tentatively attributed to a sequential crystallization process involving nucleation, growth, and transformation/additional crystallization of these phases. The endothermic event at 772 °C could reasonably be associated with the melting of the crystalline fraction developed during heating.

Sample S28 presented a *T*_g_ value of 445 °C, a single exothermic event at 544 °C, and Δ*T* = 99 °C, without resolved endothermic events within the investigated temperature range. Unlike samples S24 and S26, this composition exhibited only one dominant crystallization event, suggesting that devitrification occurred in a simpler manner or within a narrower temperature range. Given its lower CdO content and higher GeO_2_ proportion relative to those of sample S26, this response was consistent with a relatively more stable network and lower tendency to form multiple crystalline populations during heating. The peak at 544 °C could be attributed to the dominant crystallization of a phase enriched with Cd–Te–O or with a major structural rearrangement within the modified TeO_2_ network. However, without postheat treatment XRD evidence, it was not possible to distinguish with certainty between CdO, Cd_3_TeO_6_, or another TeO_2_-related phase.

Sample S30 exhibited the highest *T*_g_ value within the compositional series, at 495 °C, two exothermic events at 563 and 634 °C, and Δ*T* = 69 °C. The high *T*_g_ value indicated a relatively rigid network prior to the onset of cooperative mobility, although the moderate Δ*T* revealed that crystallization occurred without a wide thermal margin upon exceeding *T*_g_. This combination suggested that structural domains were capable of reorganizing and crystallizing in two steps despite the initial structure exhibiting thermal resistance. Owing to its high CdO content and low GeO_2_ content, the exothermic events were tentatively associated with the sequential crystallization of phases such as CdO and Cd_3_TeO_6_ or with nucleation followed by the growth of the same phase under different kinetics. The absence of a well-defined melting endotherm up to 800 °C suggested that the crystallized fraction was limited, melting occurred above the measured interval, or melting events were too broad to be resolved.

By contrast, samples S32, S34, and S36 exhibited *T*_g_ values of 445 °C, 442 °C, and 416 °C, respectively, with no detectable exothermic or endothermic peaks up to 800 °C. Under the applied heating conditions, this behavior indicated that these compositions remained predominantly amorphous and did not develop sufficiently intense crystallization to be resolved via DSC. The absence of exothermic peaks for these samples was consistent with their lower CdO contents, which reduced the probability of precipitation of Cd-rich phases. Notably, sample S36, despite exhibiting the lowest *T*_g_ value, did not display detectable crystallization. This behavior confirmed that a low *T*_g_ value did not necessarily imply poor resistance to devitrification, since the *T*_g_ and crystallization stability are related but not equivalent structural features.

Overall, the DSC results suggested that compositions with a higher CdO content, especially samples S26 and S30, exhibited the strongest tendency toward crystallization during heating, whereas samples S32, S34, and S36 demonstrated the most thermally stable behavior within the analyzed temperature range. Sample S24 displayed stepwise crystallization together with two melting events, suggesting the formation of more than one crystalline phase or phases with different stability. In turn, sample S28 exhibited a single dominant crystallization event. From a structural standpoint, this trend was consistent with the modifying role of CdO, which favored the formation of TeO_3_/TeO_3+1_ unit at the expense of TeO_4_, while GeO_2_ contributed to strengthening the glass network and improving thermal stability.

## 4 Conclusions

The glass formation region of the ternary CdO–TeO_2_–GeO_2_ glass system was systematically established using the melt-quenching method, revealing a broad compositional range in which transparent and partially crystallized glasses were obtained. Devitrification was limited to approximately 19% of the investigated compositions, primarily among CdO-rich compositions, where inverted glass behavior was consistent with the relative proportions of network formers and modifiers (i.e., CdO, TeO_2_, and GeO_2_).

Structural characterization of TeO_2_-rich glasses demonstrated that the CdO content played a decisive role in controlling structural homogeneity. XRD patterns predominantly displayed the typical amorphous bands characteristic of glassy materials, with the exception of samples S26, which exhibited a crystalline phase related to Cd_3_TeO_6_. EDS analysis confirmed Cd, Te, and Ge as the main glass constituents, with deviations from nominal compositions attributed to Te volatilization during melting (∼3.97 mol %) and minor Al incorporation from the alumina crucible. Raman and FT-IR spectroscopic results revealed the progressive depolymerization of the TeO_2_ network with increasing CdO content, evidenced by the conversion of TeO_4_ units into TeO_3_ and TeO_3+1_ structural units. Concurrently, variations in the coordination environment of Ge ions suggested the coexistence of GeO_4_ and GeO_6_ units, depending on the composition. DSC results indicated that CdO-rich compositions presented a stronger tendency toward crystallization, while TeO_2_-rich glasses remained thermally stable and predominantly amorphous up to 800 °C.

The findings of the study contribute to a comprehensive understanding of the influence of CdO on the glass formation, structural organization, and thermal behavior of the CdO–TeO_2_–GeO_2_ glass system. The provided insights highlight the potential of these glasses as thermally stable matrices, establishing a solid foundation for future investigations targeting optical and optoelectronic applications in the UV–visible and NIR spectral regions.
